# Supplementary material for: The genome sequence of a longhorn beetle, Stictoleptura scutellata (Fabricius, 1781) (Coleoptera: Cerambycidae)
Source: Wellcome Open Res. 2025 Nov 5;10:615. [Version 1] doi: 10.12688/wellcomeopenres.25051.1 (PMC12770889; doi:10.12688/wellcomeopenres.25051.1)
Supplement: Peer review history

# Reviewer report: Reviewer response for version 1

**DOI:** 10.21956/wellcomeopenres.27617.r140244

**Published:** 2026-01-05

**Authors:** Min Li

**Affiliations:** 1 Taiyuan Normal University, Jinzhong, China

This paper presents a chromosome-level genome assembly of *Stictoleptura scutellata* (Coleoptera: Cerambycidae), a key output of the Darwin Tree of Life Project. As the first genome assembly for the genus *Stictoleptura* , this study fills a critical gap in genomic resources for longhorn beetles and contributes valuable data to eukaryotic biodiversity research in Britain and Ireland. The manuscript is well-structured, methodologically rigorous, and comprehensive in presenting experimental details, results, and data availability. The assembly quality meets high standards (e.g., BUSCO completeness of 99.2%, QV score of 64.6) and adheres to the specifications of data note articles in Wellcome Open Research. Overall, the work is scientifically sound and suitable for Indexing with minor revisions.

 I suggest only a few minor changes:

1. Specify the expected release channel for annotation files (e.g., Ensembl, NCBI) in the "Data Availability" section to guide comparative genomics and functional studies.
2. Before DNA extraction, were the specimens undergone aseptic treatment?
3. Expand on population status variations across the species’ range (e.g., endangered status in different regions, major threats to habitats) to emphasize the relevance of genomic data for conservation.
4. Page 6: For the sentence “Chromosome X was assigned based on aligment to that of *Leptura quadrifasciata* (GCA_963675555.1)”, change “aligment” for “alignment”.
5. Ensure consistency in software version reporting (e.g., specify PretextSnapshot version if available, or note “unversioned” if applicable).

Are sufficient details of methods and materials provided to allow replication by others?

Yes

Is the rationale for creating the dataset(s) clearly described?

Yes

Are the datasets clearly presented in a useable and accessible format?

Yes

Are the protocols appropriate and is the work technically sound?

Yes

Reviewer Expertise:

Insect Molecular Ecology

I confirm that I have read this submission and believe that I have an appropriate level of expertise to confirm that it is of an acceptable scientific standard.

# Reviewer report: Reviewer response for version 1

**DOI:** 10.21956/wellcomeopenres.27617.r140240

**Published:** 2025-12-29

**Authors:** Christopher B. Cunningham

**Affiliations:** 1 University of Georgia, Georgia, USA

The project continues to produce quality resources for the insect genomics community. The methods are sound and relatively well documented. The article needs has enough details to be reasonably reproducible, especially now with the inclusion of the pipeline documentation. The data supporting the article are publicly available.

 Keywords. These are repeated form the title and abstract so add no value. Google indexes off these too so they should reflect some broader value.

 Background. Background. The stated motivation for this study in the introduction is fine – part of a very large survey.

 Methods. Version number for the software used is not reported. I think its reasonably reproducible but reporting that is the field standard.

 BUSCO completeness needs to be assessed with the updated OrthoDB version 12. Difference from 10 to 12 have made a big difference for several insects I work with and colleagues have reported the same. The update has been out for 13 months.

 I find it odd to produce RNA-seq data and then not do anything with it. But its generation is described reasonably enough that it could be useful for someone following up on this report.

Are sufficient details of methods and materials provided to allow replication by others?

Yes

Is the rationale for creating the dataset(s) clearly described?

Yes

Are the datasets clearly presented in a useable and accessible format?

Yes

Are the protocols appropriate and is the work technically sound?

Yes

Reviewer Expertise:

Insects, Behavior, Evolution, Genetics, Genomics, Epigenetics, Reproduction.

I confirm that I have read this submission and believe that I have an appropriate level of expertise to confirm that it is of an acceptable scientific standard, however I have significant reservations, as outlined above.

# Reviewer report: Reviewer response for version 1

**DOI:** 10.21956/wellcomeopenres.27617.r138767

**Published:** 2025-11-22

**Authors:** Peng-Yu Jin

**Affiliations:** 1 University of the Chinese Academy of Sciences, Beijing, Beijing, China; 2 Anhui Normal University School of Life Sciences (Ringgold ID: 644804), Wuhu, Anhui, China

The manuscript is excellent in describing the species, sample source, sequencing and analysis methods, results, and data availability. I have no concerns; it provides a very good reference workflow for genome sequencing and assembly. One minor point is that, as in other papers, GFF annotation results are rarely included. Providing these would be useful for future downstream analyses or comparative studies.

Are sufficient details of methods and materials provided to allow replication by others?

Yes

Is the rationale for creating the dataset(s) clearly described?

Yes

Are the datasets clearly presented in a useable and accessible format?

Yes

Are the protocols appropriate and is the work technically sound?

Yes

Reviewer Expertise:

Comparative genomics; Single-cell RNA sequencing; Animal behavior

I confirm that I have read this submission and believe that I have an appropriate level of expertise to confirm that it is of an acceptable scientific standard.
